# Equine models in translational medicine: A comparative approach to human health

**DOI:** 10.1002/ame2.70180

**Published:** 2026-03-16

**Authors:** Shayan Boozarjomehri Amnieh, Katarzyna Ropka‐Molik

**Affiliations:** ^1^ Animal Model Integrated Network (AMIN) Universal Scientific Education & Research Network (USERN) Tehran Iran; ^2^ Department of Animal Molecular Biology National Research Institute of Animal Production Balice Poland

**Keywords:** animal models, comparative medicine, equine model, One Health, translational research

## Abstract

The horse is a distinctive translational model for bridging mechanistic discovery and clinically relevant investigation because of its physiological complexity, long lifespan, athletic phenotype, and broad spectrum of naturally occurring conditions that parallel aspects of human health and disease. Its utility extends from musculoskeletal and joint research to immunology, metabolic disorders, and exercise physiology, particularly where naturally developed disease, clinically applicable imaging, and longitudinal sampling are required. Additionally, it offers opportunities to examine both chronic and acute pathological processes in a setting that closely approximates clinical reality. Advances in molecular profiling, imaging technologies, and biomarker discovery have expanded the scope of equine‐based studies, enabled refined mechanistic insights, and facilitated translational strategies. The integration of equine research into comparative medicine frameworks holds promise for accelerating therapeutic innovation within comparative and One‐Health, improving health outcomes across species.

## Graphical abstract

This diagram summarizes and contrasts rodent and equine models, outlining their strengths, limitations, and applications. Horses offer naturally occurring diseases, genetic and physiological similarities to humans, and suitability for longitudinal and clinical‐scale studies. These features make them valuable as an animal model in research, in bridging basic discoveries and clinical translation.

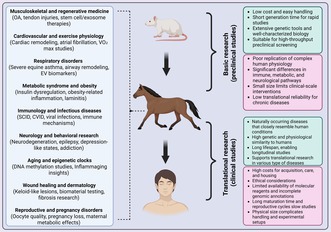

## 1 INTRODUCTION

Despite advances in drug discovery, including genomic insights and novel biologics, clinical success rates are still low, mainly because of inefficiencies in late‐stage trials. A major factor is the reliance on predictive animal models during early phases of therapeutic development. Improving target validation and designing appropriate animal models are crucial for enhancing translational outcomes and clinical success rates.^1^

On the one hand, micro‐physiological systems using human cells and organoids at the in vitro level have significantly advanced and can provide novel investigative opportunities. On the other hand, animal models can bring more comprehensive insight into the pathophysiological mechanisms underlying specific diseases, as they more closely recapitulate the complex interactions and physiological conditions occurring in vivo. However, comparable models utilizing integrated animal‐based systems have been insufficiently developed and explored in experimental research. These animal‐based systems can be essential for two key reasons: to ensure consistency with the requirements of animal toxicity studies before human trials and to differentiate species‐specific effects from those relevant to human biology.^2^ They can also help us better understand the basic biological mechanisms that occur in a given disease or under specific experimental conditions.

Regulatory organizations, including the U.S. Food and Drug Administration (FDA), increasingly support the use of animal models, particularly for complex modalities such as gene and cell therapies, where efficacy and safety are often evaluated together. To ensure their reliability in safety studies, animal models must accurately and consistently reproduce key phenotypic traits and disease progression relevant to humans. However, because no single model fully captures the complexity of human diseases, caution is essential when interpreting results. Poorly characterized or inappropriate models can lead to falsely positive results, incorrect conclusions, and the premature discontinuation of promising candidates.^3^

Traditional animal models, such as rodents and nonhuman primates, have played a crucial role in biomedical research; however, they have significant limitations in mimicking complex human diseases. Differences in genetics, immune responses, metabolism, and disease development among species often lead to poor translational relevance. These differences can lead to inaccurate predictions of drug effectiveness or safety in humans, especially for diseases involving complex immune, neurological, or metabolic processes. As a result, relying on traditional models may lead to high failure rates in clinical trials and highlights the need for more predictive, human‐centered systems.^4^, ^5^

Horses, bred mainly for athletic ability rather than for food or appearance, serve as a unique model for studying human traits such as endurance, speed, and musculoskeletal or metabolic diseases. Research focused on uncovering the genetic causes of these conditions can offer a valuable tool for understanding both equine and human health, further supporting the role of horses as a significant, unconventional model for studying human‐related disorders.^6^ More than 100 hereditary conditions in horses parallel human disorders, including inflammation, muscle and fertility abnormalities, osteoarthritis (OA), and even depression, making the horse a valuable model for studying the genetic basis, pathophysiology, and potential treatments of these diseases.^7^, ^8^, ^9^ Additionally, horses and humans share genetic similarities that lead to comparable hereditary diseases, such as Waardenburg syndrome type II and malignant hyperthermia (MH). MH triggers dangerous muscle rigidity, hyperthermia, and arrhythmias in response to certain anesthetics and occasionally to extreme exercise or heat.^10^, ^11^ Collagen‐related gene disorders highlight parallels between Fragile Foal Syndrome (FFS) in horses and Ehlers–Danlos syndromes (EDS) in humans. In warmblood horses, FFS arises from a recessive mutation in the *PLOD1* gene, leading to an EDS‐like condition characterized by fragile skin, joint laxity, and internal tissue weakness.^12^ Similarly, in humans, *PLOD1*‐associated kyphoscoliotic EDS presents with hypotonia, joint hypermobility, early‐onset kyphoscoliosis, skin fragility, and ocular abnormalities, while cognitive function generally remains unaffected. Although life expectancy can be normal, affected individuals face heightened risks of arterial rupture, spontaneous vascular dissections, restrictive lung complications, and cardiac failure in severe cases.^13^ These similarities underscore the critical role of the collagen biosynthesis pathway in maintaining connective tissue integrity across species.

This review explores the value of equine models in translational medicine by focusing on specific disease areas relevant to human health. This study aimed to assess how equine models help in understanding disease mechanisms and developing therapies. The emphasis is on their practical uses, current limitations, and prospects in comparative biomedical research.

**FIGURE 1:**
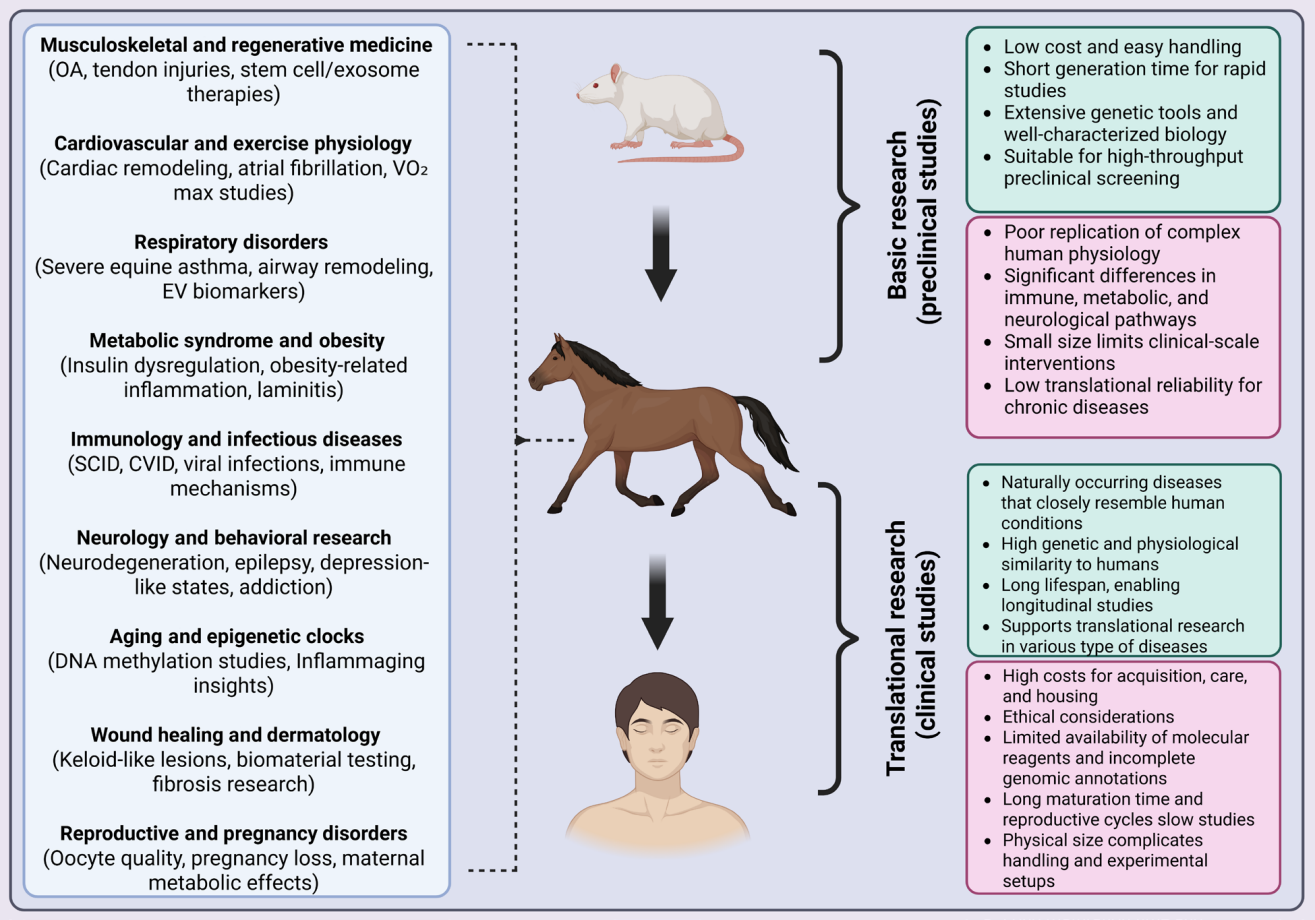
Comparison of rodent and equine models highlighting disease areas and applications.

Although the horse is increasingly recognized as a great model across diverse areas of biomedical research, its translational relevance is most pronounced in a limited number of diseases in which replacement by smaller animal models or in vitro approaches remains difficult. In particular, equine models provide clear translational value in musculoskeletal disorders, including load‐related tendinopathies and OA, in chronic airway disease associated with structural remodeling, such as severe equine asthma, and in cardiac arrhythmias (most atrial fibrillation [AF] in athletic horses) (Table 1; Figure 1). Therefore, the spontaneous nature of disease development together with clinically relevant organ size supports the strength of the equine model.

**TABLE 1:** Translational research areas currently relying on the horse model with representative studies, where direct replacement by other models is limited.

Disease area	Translational relevance of the horse model	References
Musculoskeletal disease: tendinopathy and osteoarthritis	Horses naturally develop load‐related tendinopathies and osteoarthritis under biomechanical conditions comparable to human athletes. The size of joints and tendons enables clinical‐scale imaging, arthroscopy, repeated sampling, and long‐term follow‐up, which cannot be adequately implemented based on rodents or in vitro studies	14, 15, 16, 17, 18, 19
Airway disease: severe equine asthma as a model of airway remodeling	Severe equine asthma is a naturally occurring, chronic inflammatory disease characterized by persistent airway remodeling, bronchoconstriction, and the neutrophilic inflammation. Unlike induced rodent models, it enables longitudinal assessment of structural lung changes and therapeutic responses in adult individuals	20, 21
Cardiac arrhythmias: atrial fibrillation in athletic horses	Horses spontaneously develop atrial fibrillation especially in athletic individuals with large, remodeled atria. This allows the investigation of electrical and structural atrial remodeling in a large heart under physiological and exercise‐related conditions not achievable in small‐animal models	22, 23, 24
Wound healing disorders: fibroproliferative repair	Horses are one of the few species, alongside humans, that naturally develop excessive fibroproliferative responses during wound healing. This makes them a great and unique model for studying dysregulated repair processes similar to human keloids and hypertrophic scarring, which doesn't translate well to rodent models	25, 26

Across diverse disease areas, the horse emerges as a translationally useful model primarily because many clinically relevant phenotypes arise spontaneously and can be interrogated longitudinally at a clinical scale. Unlike induced short‐term models, equine diseases often develop naturally, progress over time, and reflect real‐life mechanical, environmental, and aging‐related influences.

An important advantage of the equine model is its suitability for repeated, noninvasive, clinical‐grade investigations, including imaging, physiological monitoring, and longitudinal sampling. The large body and organ size of horses allows the wide application of diagnostic and interventional tools directly comparable to those used in human clinical practice. Such an approach enables clinically anchored study designs in a way difficult to achieve in small species.

In addition, the relatively long lifespan of horses supports long‐term follow‐up (LTFU) and the investigation of chronic disease trajectories, treatment responses, and structural remodeling processes. Together, the spontaneous nature of disease development, clinically relevant anatomy, and capacity for longitudinal assessment position the horse as a complementary late‐preclinical model that bridges mechanistic research and human clinical translation.

## 2 EQUINE‐SPECIFIC RESEARCH APPLICATIONS

### 2.1 Musculoskeletal and regenerative medicine

The horse is a valuable model for human age‐related musculoskeletal disorders because of its comparable size, natural aging process, exercise patterns, and occurrence of spontaneous injuries that resemble those in humans. Over the past decade, cell‐based regenerative treatments have been used more frequently for equine musculoskeletal diseases, offering both clinical benefits for horses and translational insights relevant to human medicine.^27^ However, despite extensive preclinical and early clinical investigation, mesenchymal stromal cell–based therapies remain associated with substantial translational challenges, including product heterogeneity, variable mechanisms of action, and limited predictability of clinical efficacy.^28^

Equine tendinopathies, particularly those involving the superficial digital flexor tendon (SDFT), closely resemble human Achilles tendon (AT) disorders, with shared genetic risk factors such as tenascin‐C and collagen V polymorphisms. Injuries to the equine SDFT serve as a well‐established model for exercise‐induced AT injury in humans, offering unique advantages over rodent or rabbit models due to their similar tendon structure, function, and response to mechanical stress. This similarity makes horses especially valuable for studying tendon maturation, aging, and the early accumulation of microdamage. Although equines primarily represent acute ruptures rather than chronic tendinopathies, their comparable longevity, lifestyle factors, and cellular stress pathways make them a highly relevant and reliable model for advancing translational tendon research.^27^ This similarity is important because there are only a few reliable laboratory models for human tendon disease. Advances in regenerative medicine, often adopted faster in equine practice owing to a more flexible regulatory environment, include non‐stem cell approaches such as scaffolds, growth factors, and platelet‐rich plasma (PRP), as well as stem cell–based therapies. PRP, which is rich in growth factors such as TGFβ‐1 (transforming growth factor beta 1), PDGF (platelet‐derived growth factor), and VEGF (vascular endothelial growth factor), has shown promise in improving tendon healing, although its impact on fibrosis remains debated.^27^ Studies have examined age‐associated changes in gene expression and collagen fiber structure and evaluated regenerative therapies, such as fetal‐derived embryonic‐like stem cells, tendon‐derived progenitor cells, and tenogenic‐primed mesenchymal stem cells, in experimentally induced tendon injuries. Comparative analyses of imaging, histology, and biomechanics have revealed the efficacy and safety of these approaches, whereas surgical interventions, including accessory ligament desmotomy, have been assessed via advanced tools such as radiofrequency probes. Large animal models, particularly horses, offer translational value because of their anatomical and functional similarities with humans and highlight the mutual benefits of equine–human comparative research, both for enhancing equine welfare and for accelerating translational progress in human regenerative therapies.^29^ The discovery that the interfascicular matrix (IFM) houses a vascular‐rich network containing CD146+ cells, endothelial cells, and mural cells provides insights into the vascular niches of tendons in both species. Although such CD146+ cells are not stem‐like, their ability to migrate to injury sites in horses mirrors repair processes in human tendons, highlighting the equine model's potential for advancing the understanding of tendon healing and developing targeted regenerative strategies in humans.^30^

OA is a chronic joint disease that has long been considered a simple wear‐and‐tear condition.^31^ The key risk factors for OA include older age, female sex, obesity, a sedentary lifestyle, and physically demanding occupations. The global burden of OA is increasing, mainly due to population aging and rising obesity rates, which strongly contribute to knee and hip OA. Although OA affects mainly weight‐bearing joints, non‐weight‐bearing joints can also be involved. The link between obesity and OA is well established, primarily because excess body weight adds mechanical stress to the joints.^32^ Previous studies support the use of horses as a model for human OA, as the mechanical forces on equine joints during exercise are similar to those in humans. This similarity in joint loading and disease process makes the One Health approach, which emphasizes the interconnectedness of human and animal health and fosters integrative, cross‐species research strategies, especially relevant for advancing OA research.^33^, ^34^

By comparing the histological and biochemical characteristics of equine and human articular cartilage from the femoral condyles, a study evaluated the translational potential of the equine model for cartilage tissue engineering. Both species exhibited similar cartilage thickness ranges, with a significant mediolateral difference in equine but not human samples, likely reflecting species‐specific joint loading patterns.^32^ Depth‐dependent distributions of glycosaminoglycans (GAGs) and DNA were observed in both species, whereas the collagen content remained consistent across layers.^35^, ^36^ These biochemical and structural similarities highlight the value of the equine model, which also offers unique advantages such as naturally occurring cartilage defects, the ability to perform second‐look arthroscopies, long‐term follow‐up, and high mechanical loading comparable to human conditions. These features make the horse a robust, large animal model for preclinical evaluation of regenerative therapies, facilitating more reliable translation to human clinical applications.^37^

The equine carpal osteochondral fragment model reliably induces OA without severe lameness, mirroring natural horse disease and providing translational relevance to human OA. It allows detailed assessment via clinical scoring, imaging, biomarkers, and tissue analysis, and has been used to test therapies such as corticosteroids, hyaluronan, PSGAG, IL‐1ra gene therapy, serum, stem cells, and pentosan. Notably, triamcinolone, hyaluronan, PSGAG, and IL‐1ra have benefits, whereas stem cells have a limited effect. Standardized lesion creation, exercise, and measures make it a robust preclinical platform, informing equine practice and human therapy.^38^, ^39^, ^40^ Articular cartilage defects, even those that are asymptomatic, accelerate loss and worsen the disease. To date, equine chondral defect models aid in the development of cartilage regeneration strategies, microfracture studies, gene therapy, stem cell studies, chondrocyte implantation, and scaffold techniques.^15^

Moreover, horse athletes are outstanding models for studying naturally occurring and posttraumatic OA, as their joints bear more than 60% of their body weight. They share similar imaging techniques and diagnostic approaches with humans, and their large size allows for relatively noninvasive arthroscopy and the collection of substantial volumes of synovial fluid for analysis.^41^

OA of the temporomandibular joint (TMJ) occurs spontaneously in both humans and horses, with the equine model offering notable translational value owing to comparable anatomical and functional features; slow age‐related disease progression; and the feasibility of repeated clinical, imaging, and tissue assessments. Recent investigations in horses have examined inflammatory mediators such as IL‐1, IL‐6, TNF‐α, TGF‐β, and PGE_2_; however, biomarkers related to cartilage degradation, chondrocyte hypertrophy, angiogenesis, and mechanical overload, as well as associated signaling pathways, remain poorly characterized. Further research focusing on cross‐species biomarker validation and mechanistic pathway analysis is warranted to strengthen the equine TMJ OA model for diagnostic, prognostic, and therapeutic applications.^42^

In turn, proteomic analyses of equine synovial fluid have revealed disease‐specific protein changes in OA, osteochondrosis, and septic synovitis, including potential biomarkers such as lacto‐transferrin, IGFBP6, S100A10, and CD109. The findings also revealed altered lubricin glycosylation, acute‐phase protein responses, and cartilage matrix degradation markers. Given the structural similarities between equine and human joints, these results hold translational value for studying and managing joint diseases in both species.^43^

In a horse model of synovitis induced by intra‐articular monoiodoacetic acid, acute inflammation lasted approximately 2 weeks, whereas markers of chronic inflammation persisted up to day 35. Even on day 42, histology revealed ongoing synovitis with osteoclast activity. Synovial fluid and tissue analyses revealed persistently elevated inflammatory biomarkers, including MMP13, ADAMTS4, RANKL, and Col1a2, compared with those in controls. These findings suggest that the MIA model reliably mimics persistent joint inflammation and that these biomarkers are valuable for evaluating the anti‐inflammatory efficacy of treatments.^44^

Another study tested a refined equine model for recurrent joint inflammation by administering three intra‐articular injections of low‐dose lipopolysaccharide (0.25 ng) at 2‐week intervals. Across eight horses, each injection produced consistent inflammatory and biomarker responses with minimal impact on welfare, and the joints fully recovered by the final assessment. Notably, MMP activity and joint circumference increased more after the second induction, whereas glycosaminoglycan responses declined, and the expression of collagen turnover markers (C2C, CPII) responded faster after the second induction. This model replicates repeated inflammatory episodes observed in conditions such as OA without causing lasting joint damage, making it a useful translational tool for evaluating therapies over extended periods.^45^

Endocrine disorders, which have long been recognized as contributors to joint pain and tendon injury in humans, are increasingly linked to similar musculoskeletal issues in horses. Comparative patterns include lameness, muscle atrophy, suspensory ligament degeneration, osteochondritis dissecans, and potentially metabolic OA, extending beyond the well‐known association with laminitis. One Health perspective highlights shared diagnostic, dietary, and management strategies while also identifying knowledge gaps, such as the safety of steroid joint injections in affected horses and the benefits of concurrent treatment for endocrine and orthopedic conditions. Insights from human–equine research can improve early injury detection, treatment, and performance outcomes in both species.^46^

Preclinical and clinical studies in horses also highlight the potential of mesenchymal stromal cell (MSCs)‐derived exosomes, particularly miRNAs, in mediating anti‐inflammatory and cartilage‐protective effects. Despite encouraging results, challenges remain regarding optimal cell source selection, standardization, and safety, warranting further research before MSC‐based therapies can become a reliable standard for OA management in equine and translational human medicine.^47^, ^48^

A study directly compared human and equine MSCs derived from adipose and tendon tissues to assess their potential for translational orthopedic research. MSCs of both species exhibited similar basic properties, including marker expression (CD29, CD44, CD90, and CD105) and the absence of hematopoietic markers, as well as comparable early‐passage proliferation, migration ability, and multilineage differentiation. Compared with human tendon‐derived MSCs, equine MSCs presented increased cell yields after tissue digestion, increased proliferation in late passages, and consistent osteogenic differentiation. Gene expression profiles differed, with human MSCs displaying higher levels of collagen IIIA1 and tenascin‐C but lower levels of decorin and scleraxis. Recent transcriptomic profiling of equine bone marrow‐derived MSCs further extends these observations by showing that differentiation toward adipogenic and chondrogenic lineages involves extensive remodeling of gene expression, with over 2300 DEGs identified in adipogenesis and more than 800 in chondrogenesis. Particularly, genes such as *CEBPA, PPARG, FABP4*, and *LEP* were markedly upregulated during adipocyte differentiation, whereas *BMP7, IHH*, and *PHOSPHO1* emerged as key drivers of chondrogenesis. These findings underscore the plasticity of equine MSCs and highlight conserved molecular pathways also implicated in human MSC differentiation, strengthening the translational relevance of the horse model.^49^ Overall, the high functional and phenotypic similarity supports the use of the horse as a valid translational model for MSC‐based orthopedic therapies, despite some species‐specific differences.^50^

Cutaneous wounds significantly impact the health and economy of both humans and horses, especially some chronic wounds that resist existing treatments. Although our understanding of wound healing has improved, traditional therapies are still mainly supportive and lack targeted solutions. Horses serve as a valuable model for wound research because of their similar skin structure and healing processes, along with the natural occurrence of difficult‐to‐heal wounds in both species. Their larger size and ease of management facilitate the collection of extensive tissue samples, increasing translational research. Both humans and horses are studied via approaches such as growth factor, cytokine, and oxygen therapy, but their clinical use is limited. The widespread and costly nature of wounds underscores the urgent need for innovative, mechanism‐based treatments to enhance healing outcomes.^25^

Another study validated adult horses as effective large animal models for testing silk‐based biomaterials for soft tissue regeneration. Both fast‐ and slow‐degrading silk matrices were implanted in subcutaneous and intramuscular sites, showing that degradation rates can be tailored for specific applications. The equine model allows multiple implant evaluations in one animal, long‐term follow‐up, ample tissue access, and noninvasive ultrasound monitoring, offering clinically relevant insights for human regenerative medicine while reducing animal use and maintaining high welfare standards.^51^ In a complementary approach, Sparks et al.^52^ demonstrated the utility of an equine distal limb wound model to study the biomechanics of tissue repair. Their work showed that wound location significantly influences mechanical properties and healing outcomes, and that treatment with a synthetic peptide modulated collagen organization and improved tensile strength.^52^ Such findings highlight the versatility of the horse not only for evaluating biomaterial degradation but also for mechanistic studies of wound repair, providing translationally relevant insights for the development of regenerative strategies in humans.^52^

Equines are a clinically relevant model for human keloids, given their shared wound‐healing characteristics and fibroproliferative pathology. A previous study linked proud flesh in equine leg wounds to micro‐vessel occlusion, hypoxia, prolonged inflammation, and profibrotic signaling. It also advances regenerative strategies by differentiating equine‐induced pluripotent stem cells into keratinocytes for potential tissue‐engineered skin substitutes, offering benefits for both equine welfare and human scar management.^26^

Within musculoskeletal research, the equine model is particularly well established for energy‐storing tendon injury and naturally occurring OA. These models allow repeated arthroscopic evaluation, clinically applicable imaging, and long‐term monitoring of tissue remodeling and therapeutic responses under physiological loading conditions, which are not feasible in rodents and remain limited in other livestock species. As such, horses provide a unique model for translational studies of disease progression and regenerative interventions.^14^, ^15^, ^16^, ^29^, ^37^

### 2.2 Cardiovascular system and diseases

Horses exhibit sport‐specific cardiac adaptations: long‐duration, high‐intensity endurance training increases the left ventricular internal diameter to meet diastolic demands. High‐power, short‐duration sports, such as jumping, cause greater wall thickness because of elevated afterload. Moderate‐intensity aerobic sports, such as dressage, increase the combined wall thickness and chamber size. Cardiac electrical activity is strongly correlated with structural changes across disciplines, especially during endurance exercise.^53^ These findings reflect human athletic patterns and highlight the importance of cardiovascular assessment in sports horse health monitoring.^53^, ^54^

Comparing myocardial structure in athletic Thoroughbred racehorses and sedentary wild horses reveals exercise‐induced cardiac remodeling. Athletic horses showed greater hypertrophy, fibrosis, and fibroblast infiltration, especially in the right atrium and ventricles, with less extracellular matrix, indicating hypertrophy at the expense of stromal tissue. These changes, which are not linked to the cause of death, result from repeated high‐intensity training, similar to that in human athletes, where stress promotes structural remodeling that can lead to arrhythmias. The right‐sided focus may reflect the pressure load during intense exercise. These findings support the use of the horse as a large animal model to study exercise‐induced myocardial changes related to arrhythmias.^55^

Moreover, several studies have evaluated chronic AF in horses as a potential large‐animal model for AF research. Compared with control horses, tachypaced horses presented an increased AF rate, decreased atrial function, and more atrial fibrosis, whereas ventricular function and ion channel expression remained the same. These results suggest that induced AF in horses leads to electrical, functional, and structural changes similar to those observed in humans, supporting their use as a relevant translational model.^22^, ^23^, ^24^, ^56^

In contrast, another study offers a comprehensive transcriptomic prediction of the equine cardiac channelome, highlighting significant differences compared with humans. Analysis of the RNA‐seq data revealed 91 predicted genes coding for ion channels, with notable findings, including the predominance of NaV1.4 over NaV1.5, high expression of the KCNA4 and KCNA7 potassium channels, and RYR1/SERCA1/CASQ1 as the main Ca^2+^‐handling proteins instead of the human‐dominant RYR2/SERCA2/CASQ2 isoforms.^57^ These differences suggest unique electrophysiological adaptations in horses, possibly linked to their high aerobic capacity and athletic performance, and may impact arrhythmia mechanisms and therapeutic responses. This work highlights the need for targeted validation studies, as equine‐specific ion channel data are essential for improving both human cardiac disease modeling and the treatment of equine arrhythmias, such as AF.^57^

Beyond physiological remodeling, horses naturally develop acquired degenerative cardiovascular conditions, including valvular insufficiency and myocardial fibrosis, which resulted from electrical instability. Importantly, ventricular arrhythmias frequently occur during and after extreme physical effort in horses, particularly premature ventricular complexes and complex ventricular ectopy.^58^

#### 2.2.1 Acquired (degenerative) cardiac disease and valve pathology

In addition to athletic remodeling and rhythm disorders, horses develop acquired cardiovascular conditions that are particularly relevant to translational cardiology because they emerge spontaneously, progress over time, and can be monitored longitudinally using clinical‐grade imaging and electrophysiology.^26^ Degenerative valvular disease, reported particularly in aging horses, represents a clinically meaningful disease spectrum in which structural valve changes, chamber remodeling, and functional consequences can be tracked under real‐life hemodynamic loading. The equine setting enables repeated echocardiographic phenotyping, assessment of exercise tolerance, and correlation of structural findings with rhythm disturbances, providing a platform for studying how chronic mechanical stress, aging, and fibrosis contribute to clinically significant remodeling.^55^ These naturally occurring features strengthen the horse's role as a complementary model for acquired cardiac disease pathways that are difficult to reproduce in short‐lived laboratory species.^59^

#### 2.2.2 Post‐exercise ventricular arrhythmias and exercise‐associated risk phenotypes

Horses provide a naturally occurring, high‐physiologic‐load model for investigating exercise‐associated ventricular arrhythmias, including post‐exercise ventricular ectopy and potentially higher‐risk ventricular rhythm phenotypes in athletic populations. Their extreme cardiopulmonary capacity, large stroke volume, and intense training exposures enable the evaluation of rhythm dynamics in conditions that approximate elite human athletics.^55^ Importantly, post‐exercise monitoring allows the characterization of the temporal relationship between exertion, autonomic rebound, electrolyte shifts, and ventricular ectopy, while simultaneously linking arrhythmic findings with structural remodeling and myocardial fibrosis phenotypes identified through imaging and tissue studies. This integrated clinical–mechanistic context supports the translational use of the horse for interrogating exercise‐triggered arrhythmia mechanisms and refining risk markers relevant to human sports cardiology.^59^, ^60^

### 2.3 Respiratory system and diseases

Asthma is a chronic airway inflammatory disease that is only partially controlled by corticosteroids and fails to reverse established lung pathology. Severe equine asthma is a naturally occurring, nonterminal disease characterized by reversible bronchospasm, Th2‐mediated neutrophilic inflammation, and lung remodeling.^20^ Cell‐based approaches, including the use of MSCs and their derivatives, have the potential to regenerate damaged tissue and reduce allergen reactivity, extending beyond the anti‐inflammatory effects of conventional treatments.^61^ In human asthma, type 2–driven inflammation is mechanistically linked to airway remodeling, including epithelial alterations, smooth muscle hypertrophy, and subepithelial fibrosis processes that may persist despite corticosteroid therapy and contribute to disease chronicity.^62^

Human asthma is a heterogeneous disorder characterized by chronic inflammation, bronchospasm, and airway remodeling, the latter contributing to progressive lung function decline. Although corticosteroids are effective against inflammation, their impact on remodeling is unclear, partly because of the lack of reliable noninvasive biomarkers. Severe equine asthma is a naturally occurring, nonterminal disease in adult horses characterized by neutrophilic inflammation, bronchospasm, mucus hypersecretion, and bronchial remodeling that persists despite remission. These features make it a valuable preclinical model for studying airway remodeling in asthma, with both similarities and differences to the human condition.^21^ The persistence of airway remodeling in severe equine asthma, including during periods of clinical remission, reflects key aspects of human asthma pathophysiology and is consistent with current known facts of type 2‐associated structural airway disease.^21^, ^62^

A study developed and validated a size‐exclusion chromatography protocol to isolate extracellular vesicles (EVs) from bronchoalveolar lavage fluid (BALF) of asthmatic and healthy horses. The findings parallel those of some human asthma studies, although methodological differences may affect particle size measurements. This work establishes horses as a natural model for studying EV biology in airway disease with translational relevance to human asthma.^63^

In the context of respiratory disease, severe equine asthma is the most extensively characterized equine model with direct translational relevance. Its naturally occurring, chronic course allows longitudinal assessment of airway remodeling, lung function, and therapeutic response in adult individuals, which cannot be reliably reproduced in induced rodent models. This makes the horse uniquely suited for studying changes associated with chronic inflammatory disease.^20^, ^21^, ^61^, ^63^

### 2.4 Metabolic syndrome and obesity

Endocrinopathies are becoming more common across different species, creating opportunities for One Health strategies to develop preventive and treatment methods, especially in athletes. In both humans and horses, obesity and the disruption of insulin and glucose metabolism affect overall health and stability. These endocrine disorders significantly impact the musculoskeletal, cardiovascular, and reproductive systems, making them particularly important for performance and long‐term health.^64^

Equine metabolic syndrome (EMS) is a growing veterinary concern closely linked to the onset of painful, often career‐ending laminitis. Subsequent studies have confirmed the complex and multifactorial nature of EMS, with clinical signs including generalized or regional adiposity, a decline in body condition score (BCS), arterial hypertension, and an increased risk of laminitis^65^, ^66^ In horses affected by EMS, adipose tissue synthesizes and secretes elevated amounts of biologically active adipokines, which dysregulate metabolic pathways, cardiovascular function, and immune responses. Affected horses commonly exhibit insulin resistance, disturbances in lipid metabolism, hyperleptinemia, and hyperinsulinemia.^67^, ^68^ Management typically involves strict diet and exercise regimens.^66^

In horses with EMS, obesity and insulin dysregulation (ID) are linked to adipose tissue dysfunction, which is characterized by adipocyte hypertrophy and macrophage infiltration, similar to humans. Differences in inflammatory cytokine expression across fat depots suggest distinct biological behaviors, supporting the role of adipose inflammation in disease progression and reinforcing the use of the horse as a model for obesity‐related research.^69^, ^70^

The value of horses as a natural model for studying noninsulin‐dependent diabetes mellitus (NIDDM) has been investigated through their analogous conditions. Unlike rodents, which do not naturally develop this disorder, horses exhibit similar age‐related weight gain, hormonal imbalances, and glucose–insulin dysregulation, as observed in humans. Research using NMR‐based metabonomics on equine plasma has identified typical and atypical metabolic responses to different dextrose doses, with findings applicable to human NIDDM. The study confirmed that EMS in horses closely mirrors human disease, making the equine model ideal for investigating metabolic pathways and pathogenesis, and that advanced analytical tools will further enhance the understanding of this condition.^71^

Notably, EMS shares many pathophysiological features with the human metabolic syndrome (MetS), including obesity, insulin resistance, dyslipidemia, hypertension, and an increased risk of secondary complications. In humans, lifestyle modifications such as increased physical activity and dietary changes can help prevent MetS and reduce associated OA symptoms. Similar benefits have been observed in horses, where exercise and diet management are key in treating EMS or ID. One emerging intervention is resistance band training, which was initially studied in humans and is now gaining popularity in equine practice.^72^ Recent research has shown that combining resistance band training with a calorie‐restricted diet improves many MetS symptoms, and even just 1 h of daily training alone produces positive effects. In horses, resistance band training has also been shown to improve thoracolumbar stability, suggesting potential benefits for overall musculoskeletal health. This approach may represent a promising therapeutic strategy across different species.^73^, ^74^

In summary, metabolic syndrome in humans mainly impacts the cardiovascular system, whereas in horses, it most often causes laminitis. Although the vascular changes behind these outcomes are not exactly the same, both species show inflammation in adipose tissue and similar changes in metabolic and biochemical processes, emphasizing shared disease features despite different clinical signs.^75^, ^76^

### 2.5 Infectious disease and immunology

Monocytes and macrophages are key components of the innate immune system, serving as the first line of defense against pathogens and playing essential roles in health and disease. Although macrophage biology has been most extensively studied in mice, the significant functional differences between mouse and human macrophages have been established.^77^ In contrast, notable similarities have been identified between equine and human macrophages, suggesting that the horse is a more comparable model than rodent, for certain aspects of macrophage function and immune research.^78^, ^79^

Foals affected by severe combined immunodeficiency (SCID), a recessively inherited genetic disorder, are unable to produce mature, functional B and T lymphocytes. This leads to a marked reduction in lymphocyte count and significantly reduced immunoglobulin levels, which progress to a complete absence of antibodies once the protection from maternal antibodies has waned. In Arabian horses, the genetic mutation responsible for SCID is relatively common, with a notable proportion of the population carrying the defective gene.^80^ Because SCID in foals closely mirrors the pathophysiology and immune dysfunction seen in human patients with the same disorder, these horses serve as a valuable translational model for studying disease mechanisms and evaluating potential therapies in human medicine.^81^

Recurrent fevers and frequent infections often prompt veterinarians to consider an underlying immune system disorder. In horses, common variable immunodeficiency (CVID) is a rare, late‐onset condition characterized by depletion or dysfunction of B lymphocytes, resulting in inadequate antibody production. Affected horses typically present with recurring respiratory tract infections (both upper and lower), meningitis with or without ataxia, cholangiohepatitis, infectious colitis, skin infections, and severe gastrointestinal parasitism. Some cases also develop immune‐mediated disorders or lymphoproliferative syndromes.^82^ Notably, equine CVID shares many clinical and immunological characteristics with the human form of the disease, making horses an important large‐animal translational model for studying disease mechanisms, testing diagnostic approaches, and exploring novel therapeutic strategies relevant to both veterinary and human medicine.^82^

It has been confirmed that the equine model is a valuable tool for studying immune responses, including the role of regulatory lymphocytes and proinflammatory cytokine expression during influenza, which presents with similar clinical signs in horses and humans. These parallels support the use of comparable vaccine development approaches in both species. Additionally, horses provide opportunities to investigate beneficial immune mechanisms and broader host‐virus interactions.^83^, ^84^

It is worth mentioning that many equine infectious pathogens share structural and genetic similarities with human pathogens, making them useful for comparative research. Notably, equine infectious anemia virus serves as a model for human immunodeficiency virus (HIV), with studies revealing a unique replication control mechanism linked to transient immunosuppression and subsequent humoral responses. Insights from this process may inform future HIV vaccine development.^85^

Moreover, horses were the first animal model used to confirm Hendra virus as the causative agent of a newly identified infectious disease. In both natural and experimental infections, the disease progresses rapidly. The natural susceptibility of horses to emerging zoonotic viruses such as Hendra and Nipah further underscores their value as translational models in One Health research, enabling the exploration of novel therapeutic and preventive strategies.^86^

Equine penile squamous cell carcinoma (epSCC), often associated with *Equus caballus* papillomavirus type 2 (EcPV‐2), has strong pathological and immune microenvironment similarities to human penile SCC caused by high‐risk HPV. In a study of 20 equine cases, 90% tested positive for EcPV‐2 DNA, with higher viral loads linked to active viral gene expression. Tumors contain abundant immune infiltrates, including T cells, macrophages, plasma cells, and regulatory T cells, often suggesting an immunosuppressive environment. These findings position epSCC as a valuable One Health translational model for human disease and highlight immune pathways that could serve as prognostic markers or therapeutic targets.^87^ The second natural model of papillomavirus‐associated tumorigenesis may be equine sarcoids induced by bovine papillomavirus types 1 and 2 (BPV‐1/2). These lesions are characterized by viral oncoprotein expression, chronicity, local invasiveness, and immune evasion, thereby resembling key features of human HPV‐driven neoplasia.^88^, ^89^ Moreover, the equine model has been successfully applied in preclinical studies of immunotherapies and vaccines, offering clinically relevant translational findings.^90^ However, unlike HPV‐associated cancers in humans, equine sarcoids rarely metastasize and are primarily driven by the viral E5 oncogene rather than E6/E7, which limits their ability to fully recapitulate the molecular mechanisms of human disease.^91^

Horses serve as a valuable natural model for autoimmune uveitis through equine recurrent uveitis (ERU), which closely mirrors human disease in terms of immunopathology and physiology. One study examined the molecular mechanisms involved in spontaneous uveitis, identifying changes in matrix enzymes and protein–protein interactions that could be targeted to potentially reverse symptoms.^92^ In parallel, another study investigated retinal glial cell function in the ERU, providing the first report of aquaporin protein AQP5 involvement in a retinal disease. These findings link breakdown of the blood–retinal barrier, T‐cell–mediated cytokine release, and reduced AQP5 expression, offering new insights into disease mechanisms. These discoveries, made possible by the equine model, open avenues for targeted therapies that might not have been identified via other models.^93^

ERU, the leading cause of blindness in horses, is a chronic, relapsing inflammatory disease of the uveal tract with multifactorial etiology that is most commonly linked to *Leptospira* infection and genetic predisposition.^94^ ERU shares striking similarities with human autoimmune uveitis, including comparable autoantigens and a remitting–relapsing disease course, making the horse the only known natural large‐animal model for the human condition. Understanding the autoimmune mechanisms of ERU, including molecular mimicry, bystander activation, and epitope spreading, is critical for developing targeted therapies beyond current symptomatic treatments such as corticosteroids, mydriatics, and vitrectomy.^94^

Studies using reproducible equine models of acute inflammation have shown that NSAIDs, by inhibiting eicosanoid synthesis, also reduce inflammatory heat, highlighting their role in acute inflammation. Enolic and carboxylic acid NSAIDs tend to accumulate in inflammatory exudates, prolonging their inhibitory effect on eicosanoid production, which is consistent with clinical observations. Dual cyclo‐oxygenase/lipoxygenase inhibitors (BW540C) and corticosteroids such as betamethasone and dexamethasone have also been tested, revealing notable and sometimes unexpected results.^95^

Studies increasingly indicate that macrophages vary considerably on the basis of their tissue of origin, challenging the idea that all macrophages share the same functions. In horses, although transcriptomic data remain limited, they suggest tissue‐specific gene expression patterns similar to those observed in other species. Advances in equine genomics and tissue biobanking have enabled more in‐depth studies of equine macrophage biology. Owing to their physiological similarities to humans and the availability of extensive tissue samples, horses hold significant promise as translational models for examining the roles of innate immunity and macrophages.^96^

### 2.6 Neurology and behavioral research

Neurological disorders (NDs) in horses, ranging from neuroinflammatory to neurodegenerative conditions, often cause severe or fatal outcomes and present diverse clinical signs depending on the affected nervous system region. Common examples include cervical vertebral stenotic myelopathy, equine degenerative myeloencephalopathy, equine motor neuron disease, and equine nigro pallidal encephalomalacia, some of which share striking pathological similarities with human diseases such as amyotrophic lateral sclerosis and Parkinson's disease. Diagnosis typically relies on clinical evaluation, differential exclusion, and ancillary tests, whereas advanced imaging remains limited by cost and accessibility. Comparative research highlights the potential of human neuro‐biomarkers, such as neurofilaments, S100B, the tau protein, and GFAP, for early detection in horses. However, biomarker‐based diagnostics are still in early development for veterinary medicine and hold promise for improving the accuracy and timeliness of ND diagnosis.^97^

Equine pituitary pars intermedia dysfunction (PPID) in aged horses shares similar pathophysiological features with Parkinson's disease, including increased α‐synuclein (α‐syn) levels. In horses with PPID, α‐syn from the pars intermedia showed prion‐like seeding properties, and fibrils were found only in affected animals. Bioinformatic and biophysical studies revealed that the 62–87 region of both equine and human α‐syn is highly prone to aggregation, with peptides from both species forming mature fibrils. These results demonstrate α‐syn misfolding in horses with PPID, highlighting their potential as a model for α‐synucleinopathies.^98^ Both horses and humans can be affected by neurodegenerative diseases, resulting in degeneration of Purkinje cells in the cerebellar abiotrophy (CA). Whereas in horses, CA is determined by a recessive inheritance background,^99^ in humans, CA has still been considered a non‐hereditary degenerative ataxia of unknown etiology.^100^ The degeneration of Purkinje cells in horses leads to ataxia and coordination deficits, closely resembling analogous human neurodegenerative diseases. Despite phenotypic similarities, the equine model can offer unique advantages for translational research: affected horses are large animals with a long lifespan, enabling longitudinal monitoring of disease progression, advanced neuroimaging, and electrophysiological assessments that are directly comparable to those used in human patients. Moreover, the availability of genetic testing for CA‐associated mutations in horses provides an opportunity to study genotype–phenotype correlations, explore early biomarkers of neurodegeneration, and evaluate novel therapeutic approaches in a physiologically and anatomically relevant system.

Juvenile idiopathic epilepsy (JIE) in Egyptian Arabian foals^101^, ^102^ is characterized by focal epileptic discharges (EDs) originating at the central vertex, often spreading to the centroparietal or frontocentral regions, and resulting in generalized tonic–clonic seizures with facial motor activity and loss of consciousness. Findings indicate that EEG with photic stimulation is valuable for precise epilepsy phenotyping and that JIE, which is benign and self‐limiting, may serve as a naturally occurring model for self‐limited childhood epilepsy.^103^

Depression‐like states in horses can be identified through the co‐occurrence of specific species‐appropriate biomarkers, reflecting findings from human and other animal studies. Key indicators include sustained inactivity, reduced reactivity to environmental stimuli, anhedonia, alterations in sleep patterns, and diminished cognitive attention. These behavioral and physiological changes are associated with poor health and welfare and may also negatively affect performance and productivity in domestic horses. Establishing baseline parameters and threshold criteria for these biomarkers, validating a multi‐biomarker approach, and developing practical field protocols are essential next steps. Such advancements would enable accurate identification of the equine depression phenotype, supporting improved welfare assessment and targeted interventions.^104^ Like in humans, these states in horses can arise from a combination of genetic predispositions and environmental stressors. If validated, the equine depression phenotype could serve as a naturally occurring large‐animal model for studying the neurobiology, physiology, and treatment responses associated with MDD.^104^, ^105^

Horses present physiological and behavioral traits that parallel human conditions, making them a promising model for investigating the genetic and neurobiological mechanisms underlying disorders such as addiction. Central dopaminergic tone, which influences traits such as impulsivity and compulsivity in humans, can also be assessed in horses through measures such as the spontaneous blink rate (SBR). Evidence suggests that SBR in horses is correlated with impulsivity, highlighting shared pathways between equine and human dopamine‐related behavior. These similarities support the use of horses to explore how genetic factors and environmental influences interact to shape addiction‐related phenotypes, offering translational value for human health research.^106^

### 2.7 Aging

Aging involves progressive physiological decline that affects multiple systems and is shaped by biological and environmental factors. What is important is, based on DNA methylation analysis, that epigenetic clocks have been established as robust biomarkers of aging in both humans and horses. Importantly, the investigation of profiles of conserved CpG sites provides highly accurate estimates of chronological age across horses and humans, with correlation coefficients approaching 0.98.^107^ Key molecular pathways, such as the mTOR and sirtuin pathways, have been linked to lifespan extension in animal models. Rodents are widely used in such investigations because of their low cost, whereas primates are more similar to humans but are expensive.^108^ However, rodents are not optimal models for aging research, as they differ substantially from humans in lifespan, body size, metabolic rate, and many aspects of age‐related pathophysiology. Their relatively short lifespan compresses the temporal dynamics of aging, limiting the ability to capture gradual, cumulative processes observed in humans. Furthermore, rodents do not naturally develop several chronic degenerative diseases commonly associated with human aging, such as spontaneous OA or neurodegenerative disorders, which reduces their translational relevance.^109^ Horses share similar body structures, aging patterns, and naturally developed diseases with humans, including conditions such as diabetes and polysaccharide storage myopathy. The relatively large aging population enables studies on age‐related pathologies that progress similarly in both species, creating opportunities for mutual therapeutic advancements.^108^

The increasing number of geriatric horses worldwide highlights the need to better understand equine aging, especially its impact on immunity, the vaccine response, and disease susceptibility. Aging horses show immune changes similar to but milder than those in humans, including decreased lymphocyte proliferation, antibody production, and vaccine effectiveness. Although they display a proinflammatory state (“inflammaging”), they experience a lower rate of age‐related diseases common in humans, such as cancer, cardiovascular issues, and neurodegeneration. This resilience might result from factors such as healthier lifestyles, diet, exercise, genetics, and physiology, providing useful comparative insights for human aging research.^110^

A study has created precise DNA methylation–based age estimators (epigenetic clocks) for horses using conserved genomic sites, enabling accurate age prediction across the lifespan and in multiple equid species, including a shared human–horse clock. These tools reveal that age‐related methylation changes in horses often reflect those in humans, especially in developmental gene regions and specific chromatin states, where methylation can either suppress or promote gene expression depending on context. These findings strengthen the value of the horse as a model for studying aging biology and indicate the way for cross‐species research and future studies that could assess disease and mortality risk.^107^

### 2.8 Exercise physiology and endurance adaptation

Horses engage in intense athletic activities, making them valuable models for studying exercise physiology and immune responses relevant to humans. One of the most important arguments for using the horse as a model of exercise physiology is its extremely high maximal oxygen uptake (VO_2_ max). Elite Thoroughbred racehorses can reach values of 120–180 mL O_2_/kg/min, several‐fold higher than the maximal values observed in top human endurance athletes (70–85 mL O_2_/kg/min).^111^ This difference is largely due to horses' extraordinary cardiac output, increased ventilatory capacity, and unique splenic release of erythrocytes during exercise. Nevertheless, the underlying adaptive mechanisms, such as cardiac remodeling, mitochondrial biogenesis, and transcriptional regulation of metabolic pathways, are qualitatively similar to those in humans.^112^, ^113^ This makes the horse a powerful translational model for understanding universal processes driving aerobic and anaerobic training adaptations. Performance horses, particularly those involved in racing, also serve as natural models for exercise‐associated clinical problems that are highly relevant to human sports medicine. A notable example is exercise‐induced arrhythmias and sudden cardiac death, phenomena reported in both equine and human athletes^59^, ^60^, ^114^. Comparative cardiology studies demonstrate overlapping features, including structural remodeling, conduction abnormalities, and risk factors for rhythm disturbances. Similarly, skeletal muscle damage, electrolyte imbalances, thermoregulatory strain, and immune responses to strenuous exercise occur in both species. These parallels highlight the translational potential of equine studies for investigating mechanisms of exercise‐related pathology. Taken together, the very high VO_2_ max and the occurrence of exercise‐related clinical issues similar to those in humans make the horse a valuable translational model. At the same time, species‐specific features such as splenic erythrocyte release mean that results must be interpreted with caution when applied to humans.

Additionally, research has shown parallels in post‐exercise changes in cytokine levels, immune cell activity, and receptor expression between horses and human athletes, supporting their use in investigating the immune effects of intense physical activity.^115^ Endurance exercise in horses triggers reproducible changes in genes such as IL1B, IL1R2, and IL22RA2, along with other cytokines such as IL6 and IL8, reflecting metabolic adaptation to sustained activity. These markers, combined with hormonal measures such as testosterone–cortisol ratios, could help assess stress resistance, optimize training, and prevent overtraining syndrome. Although horses exhibit unique adaptations to intense exercise, their value as endurance models for humans remains uncertain due to physiological differences.^116^ Moreover, a recent study concerning endurance horses (endurance‐trained Arabian horses) indicated that horses can be a valuable model for validating overtraining biomarkers during endurance‐type exercise, also in humans. The pronounced changes in expression of KL (Klotho) and *ACTN3* gene isoforms following a 120 km endurance ride highlight their potential utility as molecular markers of physiological strain in prolonged aerobic exertion.^117^ In humans, the modifications of the *ACTN3* gene expression under exercise were confirmed by different training conditions.^118^

### 2.9 Reproductive and pregnancy disorders

Reproductive aging and assisted reproduction are gaining importance in human medicine, yet research focusing on women remains limited, highlighting the need for animal models. The mare, despite being underused, exhibits key physiological similarities to women, such as comparable cyclic and hormonal changes with age, patterns of increased gonadotropin, and a gradual decline in oocyte quality. In both species, oocytes remain in meiotic arrest for decades, and decreased oocyte quality significantly contributes to age‐related infertility. The mare's extended follicular phase, presence of a single dominant follicle, and similar ovulation timeline to that of women make it a valuable model for investigating reproductive aging and oocyte–follicular maturation.^9^

Reproductive aging in mares and women results in similar declines in fertility, with oocyte donor age strongly linked to reduced oocyte quality. Age‐related changes include altered morphology, gene expression, and developmental potential, with oxidative stress and mitochondrial dysfunction as key factors. In women, aneuploidy is a primary concern of age‐related subfertility and miscarriage, whereas in mares, chromosome misalignment has been observed but is less studied. The mare offers potential as a research model to investigate factors influencing oocyte quality and developmental potential in human reproductive aging.^119^ In vitro matured oocytes from aged mares presented decreased expression of the centromere cohesion–stabilizing protein Shugoshin‐1, increased aneuploidy, premature sister chromatid separation, and weakened centromeric cohesion. These findings support the use of the mare as a more appropriate model than rodents, as aged mares exhibit reduced fertility and greater early pregnancy loss, indicating that age‐related cohesion loss is a mechanism that likely contributes to embryonic aneuploidy and reduced fertility in both species.^120^

Compared with other species, the equine fetus depends more heavily on placental transfer of glucose and nutrients because of limited deamination and gluconeogenesis. This reliance suggests that the placenta may play an even greater role in fetal pathologies linked to EMS. Such early‐life programming could predispose offspring to long‐term insulin resistance, increasing the risk of juvenile and adult obesity and EMS, as observed in humans.^121^ In mature horses, obesity reduces exercise capacity and performance, increases joint strain, and is associated with increased rates of developmental orthopedic disease in growing animals. The elevated risk of EMS creates a self‐perpetuating cycle that exacerbates the condition's prevalence and amplifies its economic impact on the equine industry.^121^

Another study explored how maternal EMS impacts foal health, drawing parallels to gestational diabetes in humans. As EMS becomes more common in broodmares, concerns about its effects on placental function, fetal development, and newborn health are increasing. Early findings revealed placental histological changes in EMS‐affected mares that are similar to those observed in women with gestational diabetes, indicating a shared underlying mechanism. This research positions the horse as a useful model for studying maternal insulin issues and their effects on offspring, which could help improve management and treatment strategies for both horses and humans.^122^

Furthermore, the mare can be considered a valuable large‐animal model for studies on endometrial pathology, as equine endometriosis shares key mechanisms with human endometriosis, including chronic inflammation, macrophage activity, and progressive fibrosis of the endometrium.^123^ Its main advantages are the spontaneous nature of the disease (not artificially induced as in rodents), the similarity of the reproductive cycle to that of women, and the accessibility of tissue for translational analyses. However, the model also has limitations, as lesions in mares are confined to the uterine lining rather than ectopic sites, and thus cannot fully reproduce the clinical complexity and pain‐associated symptoms of human endometriosis.

Chromosomal abnormalities are a leading cause of miscarriage in humans, but are rarely studied in animals because of the limited number of available models. Horses, with their high level of reproductive monitoring in mares, present a promising natural model. In a study of 256 equine pregnancy losses, chromosomal copy number abnormalities were found in more than half of the cases, with triploidy being the most common. The similarities between equine and human pregnancy, including comparable gestation lengths, age‐related pregnancy loss, and chromosomal synteny, emphasize the horse as a valuable model for studying miscarriages caused by chromosomal abnormalities.^124^

Equine somatic cell nuclear transfer (SCNT) can be effectively applied in commercial breeding to produce healthy cloned foals via either abattoir or ovum pick‐up (OPU)‐derived oocytes, with OPU being markedly more efficient. Vitrification of OPU‐derived blastocysts does not impair developmental potential and enables embryo transfer to coincide with optimal uterine receptivity. These findings emphasize the value of OPU‐derived oocytes for improving cloning efficiency and position the horse as a valuable model for advancing reproductive and developmental biology.^125^

Despite notable differences in reproductive anatomy, physiology, and assisted reproductive technology (ART) between horses and humans, both face infertility issues related to aging and obesity. Although some ART techniques are more advanced in humans (e.g., superovulation and cryopreservation), others, such as in vitro maturation, have shown greater advances in horses. Comparative studies can provide useful insights for both species, even though horses are not direct models for human ART. Interestingly, early embryonic development stages, such as meiosis resumption, pronuclei formation, cleavage timing, and morula formation, are remarkably similar between women and mares until the blastocyst stage, which occurs slightly later in horses.^126^

## 3 STRENGTHS, BARRIERS, AND STRATEGIES FOR BROADER INTEGRATION

Advances in regenerative medicine face significant obstacles in translating small animal research into large animal models that are clinically relevant. Selecting the right model requires a thorough understanding of species‐specific traits to ensure that the disease closely mimics human pathology. Traditional reductionist models often fall short of replicating complex conditions, creating gaps between preclinical and clinical outcomes. A stepwise approach, starting with in vitro, ex vivo, and in silico studies and then progressing to large animals with a naturally developed disease, can enhance relevance and reliability. These models not only push forward human medicine but also benefit veterinary patients, making them valuable for both research and clinical practice.^127^

The important matter is that the equine model provides a strong translational advantage as many diseases of interest arise spontaneously and occur naturally in both horses and humans. This makes it a more faithful and ethically favorable model, allowing studies that can directly inform better prevention, diagnosis, and treatment strategies to improve health and quality of life across species (Figure 1).

Drug and medical device development is costly and time‐intensive, with high clinical failure rates despite preclinical success, often owing to limitations of rodent and in vitro models that cannot replicate complex human physiology or support clinical‐scale procedures. Large animal models, including pigs, sheep, goats, and horses, offer greater anatomical, physiological, and immunological similarity to humans, enabling more translatable results for diverse disease areas despite higher maintenance costs and facility needs.^128^

Moreover, horses influence human and environmental health through ecosystem impacts, economic and therapeutic roles, and historical medical contributions. They are also linked to zoonotic and noncommunicable diseases, with shared risks such as climate change and antimicrobial resistance. Integrating horses into One Health strategies is essential for sustainable health outcomes.^129^

Unlike mice, whose innate immune responses differ markedly from those of humans, equine macrophages and monocytes exhibit functional parallels to human cells, including comparable sensitivity to lipopolysaccharide (LPS) and similar patterns of LPS‐induced gene expression. These traits are particularly relevant for studying endotoxemia, sepsis, and lung inflammation, where rodent models have shown limited translational success.^87^ As humans do, horses do not produce nitric oxide via inducible NOS in response to LPS; instead, they rely on alternative metabolic pathways, unlike murine macrophages.^130^, ^131^

Genetically, horses share a high degree of genome synteny and gene sequence homology with humans, surpassing that of rodents. Over 130 equine hereditary diseases^132^, ^133^ and even their behavioral conditions have human counterparts.^96^ Conserved immune genes such as IL2, IL17, IL23, CXCL9, CD14, and multiple TLRs reinforce this genomic proximity.^134^, ^135^, ^136^, ^137^

As discussed earlier, many horse breeds present high rates of inherited Mendelian disorders, including Fell pony syndrome, hyperkalemic periodic paralysis, and lavender foal syndrome. When combined with whole‐genome sequencing data from healthy horses, sequencing only a few affected individuals can help pinpoint causal mutations for these rare monogenic conditions. Identifying such variants not only benefits equine health but also aligns with the broader aim of using naturally occurring animal models to advance human medicine. As part of a One Medicine approach, understanding which genes can or cannot tolerate loss‐of‐function or damaging variants across species enhances disease‐gene discovery accuracy and increases the translational relevance of equine models.^6^

The equine embryo serves as a valuable model for gene‐editing research because of its high molecular similarity to humans, particularly in the pluripotency gene OCT4.^128^ Unique species‐specific expression patterns suggest that the uterine environment influences OCT4 regulation. Although CRISPR editing via somatic cell nuclear transfer has been achieved, direct zygotic editing remains unrealized. These parallels position horses as promising models for developing and refining gene therapy approaches for heritable diseases in both species.^138^

Although the equine model offers valuable research potential, it presents notable challenges, including high costs for acquisition, care, and housing, as well as ethical considerations. The limited availability of reagents, incomplete genomic and tissue‐specific annotations, long maturation times, and the physical size of horses further restrict certain types of studies.^139^, ^140^

Selecting an optimal animal model is crucial for ensuring the successful translation of research findings into human preclinical applications. In addition to functional similarities, the anatomical and histological characteristics of each species play a decisive role in determining its suitability for specific research goals.^141^ A thorough understanding of these unique features enables researchers to make informed choices, thereby increasing the likelihood of obtaining clinically relevant and translatable results.

## 4 CONCLUSION

Across the literature, the horse emerges as a translationally useful model primarily because many clinically relevant phenotypes arise spontaneously and can be interrogated longitudinally at a clinical scale. The strongest and most consistently supported applications include musculoskeletal disease, notably OA, cartilage defects, and tendon injury models with human‐comparable loading and imaging, respiratory disease, such as naturally occurring severe equine asthma with airway remodeling, and high‐physiologic‐load cardiovascular investigation, including athletic remodeling and rhythm phenotypes, with growing relevance for exercise‐associated arrhythmia research. Additional areas such as immune dysregulation and naturally occurring autoimmune diseases like equine recurrent uveitis, reproductive aging, and aging biology supported by cross‐species epigenetic clocks further extend equine value within comparative medicine and One Health. At the same time, limitations including cost, infrastructure requirements, and reagent constraints mean that equine studies are best positioned as a complementary late‐preclinical or clinically anchored platform rather than a universal substitute for rodent or in vitro systems. Strategically integrating equine evidence where its features are difficult to replicate, clinical‐scale procedures, long‐term sampling, and naturally developed disease trajectories can strengthen target validation and improve translational confidence for interventions intended for human and veterinary patients.

## AUTHOR CONTRIBUTIONS

**Shayan Boozarjomehri Amnieh:** Conceptualization; investigation; visualization; writing – original draft; writing – review and editing. **Katarzyna Ropka‐Molik:** Conceptualization; funding acquisition; supervision; validation; writing – original draft; writing – review and editing.

## FUNDING INFORMATION

The study was supported by the statutory activity of the National Research Institute of Animal Production (grant no. 04‐18‐20‐21).

## CONFLICT OF INTEREST STATEMENT

The authors declared no competing financial or commercial interests.

## ETHICS STATEMENT

None.

## Data Availability

No datasets were generated or analyzed during the current study. Therefore, no data are available.
